# Characterization of the Probiotic Yeast *Saccharomyces boulardii* in the Healthy Mucosal Immune System

**DOI:** 10.1371/journal.pone.0153351

**Published:** 2016-04-11

**Authors:** Lauren E. Hudson, Courtney D. McDermott, Taryn P. Stewart, William H. Hudson, Daniel Rios, Milo B. Fasken, Anita H. Corbett, Tracey J. Lamb

**Affiliations:** 1 Department of Pediatrics, Emory University School of Medicine, Atlanta, GA, United States of America; 2 Department of Microbiology and Immunology, Emory University School of Medicine, Atlanta, GA, United States of America; 3 Department of Pathology and Laboratory Medicine, Emory University School of Medicine, Atlanta, Georgia, United States of America; 4 Department of Biochemistry, Emory University School of Medicine, Atlanta, GA, United States of America; CNR, ITALY

## Abstract

The probiotic yeast *Saccharomyces boulardii* has been shown to ameliorate disease severity in the context of many infectious and inflammatory conditions. However, use of *S*. *boulardii* as a prophylactic agent or therapeutic delivery vector would require delivery of *S*. *boulardii* to a healthy, uninflamed intestine. In contrast to inflamed mucosal tissue, the diverse microbiota, intact epithelial barrier, and fewer inflammatory immune cells within the healthy intestine may all limit the degree to which *S*. *boulardii* contacts and influences the host mucosal immune system. Understanding the nature of these interactions is crucial for application of *S*. *boulardii* as a prophylactic agent or therapeutic delivery vehicle. In this study, we explore both intrinsic and immunomodulatory properties of *S*. *boulardii* in the healthy mucosal immune system. Genomic sequencing and morphological analysis of *S*. *boulardii* reveals changes in cell wall components compared to non-probiotic *S*. *cerevisiae* that may partially account for probiotic functions of *S*. *boulardii*. Flow cytometry and immunohistochemistry demonstrate limited *S*. *boulardii* association with murine Peyer’s patches. We also show that although *S*. *boulardii* induces a systemic humoral immune response, this response is small in magnitude and not directed against *S*. *boulardii* itself. RNA-seq of the draining mesenteric lymph nodes indicates that even repeated administration of *S*. *boulardii* induces few transcriptional changes in the healthy intestine. Together these data strongly suggest that interaction between *S*. *boulardii* and the mucosal immune system in the healthy intestine is limited, with important implications for future work examining *S*. *boulardii* as a prophylactic agent and therapeutic delivery vehicle.

## Introduction

Use of viable microorganisms to synthesize and deliver therapeutics directly to the mucosa is an intriguing potential means of treating and preventing gastrointestinal disorders. Numerous studies have investigated the use of probiotic bacteria for the delivery of gastrointestinal therapeutics; however, eukaryotic probiotics have been less well studied. A major advantage of using probiotic yeast for this application is their ability as eukaryotes to create post-translational modifications that might enable expression of a wide variety of therapeutic proteins in their proper conformation. A limited number of *Saccharomyces cerevisiae* strains, particularly *S*. *cerevisiae* subspecies *boulardii* isolates, have been identified as candidates for this novel therapeutic approach due to their ability to easily express heterologous antigen as well as their current use in treatment of gastrointestinal disorders [1,2].

*S*. *boulardii* probiotic yeast isolates have already been extensively studied in terms of their ability to limit inflammation and infection in the gastrointestinal tract [3]. However, there is currently a paucity of information regarding the effects of *S*. *boulardii* in the healthy, uninflamed intestine. Effects of probiotics observed in the context of inflammation or dysbiosis are likely to be heavily influenced by intestinal barrier breakdown and increased exposure of probiotics to host cells, increased recruitment of inflammatory immune cells to the intestine, or interactions of probiotics with an altered microbiota composition [4]. Use of *S*. *boulardii* in oral vaccine delivery or prophylaxis entails administration to the healthy host mucosa. The tolerogenic nature of the healthy intestine may affect not only the level but also the nature of the interactions between probiotics and the host. The extent of these interactions will have significant implications for the design and dosing of engineered probiotic yeast for use in disease prevention, making it crucial to understand the interactions of *S*. *boulardii* with the healthy host mucosa in the absence of infection or inflammation.

In the healthy intestine, microorganisms and antigens are largely sequestered within the center of the lumen, separated from the intestinal epithelium by thick layers of mucus, antimicrobials, and antibodies [5,6]. In order for *S*. *boulardii* to successfully deliver therapeutic proteins to the mucosal immune system, it must overcome these barriers and reach antigen-sampling cells along the epithelial layer. Goblet cells and dendritic cells (DCs) take up small particles from the intestinal lumen [7,8]; however, the host cells most likely to take up large particles such as intact yeast are the microfold (M) cells of the small intestinal Peyer’s patches (PP). These cells transcellularly transfer antigen from the intestinal lumen to the PP dome, where numerous antigen presenting cells can take up antigen and induce local immune responses as well as traffic to the draining mesenteric lymph nodes (MLN) to stimulate further responses [9]. However, contact with these antigen sampling sites may risk the induction of immune responses against *S*. *boulardii* itself. Such immune responses could sequester and clear subsequent incoming yeast or risk induction of gastrointestinal inflammation upon repeated administration.

Immune recognition of *S*. *boulardii* is most likely mediated by the cell wall, a highly complex structure that mediates responses to external stresses including anaerobic conditions as well as pH and osmotic changes [10–12]. The cell wall contains many immunomodulatory components. Mannoproteins, for example, compose the outer layer of the yeast cell wall and bind galectin 3, DC-SIGN, TLR4, and others [13]. β-glucans, which constitute the middle layer, ligate Dectin-1 and TLRs 2 and 6 and can stimulate Langerin positive DCs in small intestinal Peyer’s patches [13]. Chitin, a minor component of the innermost cell wall layer, binds the mannose receptor [14–16]. Indeed, administration of yeast cell wall fragments such as β-glucans has been found to stimulate mucosal immune responses and recapitulate some effects of whole probiotics [17–19].

Previous reports of secretory IgA induction after *S*. *boulardii* administration [20–22] suggest that *S*. *boulardii* might induce adaptive immune responses. However, there have been no reports measuring *S*. *boulardii*-induced changes in healthy systemic antibody levels or anti-*S*. *boulardii* antibodies in specific-pathogen-free (SPF) mice. Furthermore, few studies have examined cell signaling pathways and cytokines induced by *S*. *boulardii* in the healthy intestine. The goal of the present study is thus to elucidate intrinsic and immunomodulatory properties of the probiotic yeast *S*. *boulardii* in the healthy intestine. A thorough understanding of these interactions is crucial as they may affect functions of *S*. *boulardii* in prophylaxis and as a delivery vector for therapeutics to the healthy gastrointestinal tract. Our results indicate that *S*. *boulardii* has a limited ability to induce immune responses in the healthy mucosa. This suggests that observed prophylactic effects of administration of this probiotic yeast are not mediated via effects on the mucosal immune system.

## Materials and Methods

### Yeast Strains

*S*. *boulardii* (Ultra Levure®, American Type Culture Collection® Number: MYA-797™) was used in all imaging, *in vitro*, and *in vivo* studies. *S*. *cerevisiae* W303 and BY4741 are well characterized laboratory haploid strains (http://yeastgenome.org/) used in EM imaging.

### Yeast Genomic Sequencing and Analysis

Yeast genomic DNA was prepared using the ZR Fungal/Bacterial DNA MiniPrep kit (Zymo Research). Sequencing was performed by the Emory University Genomics Core on an Illumina HiSeq 2000 with 100 bp paired end reads. Velvet (version 1.2.10) was used for *de novo* assembly of contigs. The *S*. *boulardii* ATCC MYA-797 draft genome has been submitted as an NCBI Whole Genome Shotgun (WGS) project under accession number LRVB00000000. SyMap was used to detect synteny between the sequenced *S*. b*oulardii* draft genome and the *S*. *cerevisiae* reference genome (R-64-1-1, accessed via *Ensembl* [23]). SyMap and MUSCLE were used to generate the three-way alignments between the contigs reported here, the *S*. *cerevisiae* reference genome, and the previously reported *S*. *boulardii* EDRL genome [24,25]. Gene ontology enrichment was performed at the *Saccharomyces* genome database (http://www.yeastgenome.org/) [26].

### Yeast Cell Wall Analyses

Yeast were grown to saturation in normal YPD media (1% yeast extract, 2% peptone, 2% glucose/dextrose in distilled water), cryopreserved according to standard protocols and imaged using a Hitachi H7500 TEM by the Emory Robert P. Apkarian Integrated Electron Microscopy Core. Cell wall layers were measured using Image J software, taking the average measurements of 23 cells per strain. Statistics were calculated using GraphPad Prism 6 software and the Kruskal-Wallis and Dunn’s multiple comparisons tests. For caspofungin assays, yeast grown overnight in normal YPD media were diluted to 10^7^ cells per 200 μL in YPD media adjusted to acidic (pH 4) or basic (pH 8) conditions and containing a 0, 2, 4, or 6 nM concentration of caspofungin diacetate (Sigma). Control yeasts were also grown in untreated media at approximately pH 6, and assays were performed in triplicate. OD_600_ readings were taken over 24 hours incubation at 37°C. The phenol sulfuric acid assay was used to determine relative concentration of total cell wall monosaccharide content of 10^9^ yeast grown to saturation in either normal YPD media or media containing 6 nM caspofungin as previously described [27,28].

### Animal studies

Female C57BL/6 mice aged 4–6 weeks were obtained from Jackson Laboratories and maintained in sterile housing conditions. Studies were conducted according to the Guide for the Care and Use of Laboratory Animals of the National Institutes of Health and with the approval of the Emory University Institutional Animal Care and Use Committee (protocol number 2002655). For experiments with fluorescently-labeled *S*. *boulardii*, mice were gavaged as described [29] with 10^8^ CFU of carboxyfluorescein succinimidyl ester (CFSE) surface-labeled *S*. *boulardii*, and PP were harvested 0, 0.5, 1, or 2 hours later. Treatment groups in subsequent experiments were gavaged daily with 10^8^ CFU of *S*. *boulardii* resuspended in 100 μL sterile 1X PBS (Life Technologies), while naïve controls were gavaged with an equal volume of sterile PBS. Blood samples were collected by cheek bleeds into heparinized tubes and spun at 17,000 x g in a microcentrifuge for 5 min at 4°C to collect serum. Fresh fecal pellets were collected, weighed, and resuspended in 10 fold w/v PBS 2 mM EDTA containing a 1:100 dilution of the P8340 protease inhibitor (Sigma) by vortexing until homogenized. Fecal material was then pelleted by centrifugation at 17,000 xg for 10 min at 4°C and the supernatant collected. Fecal supernatant and serum were stored at -20°C. Mice were euthanized using isoflurane at the time points indicated and every effort was made to minimize suffering. Further reagent details are listed in S1 Table.

### Immunohistochemistry

Mice were gavaged with 10^8^ CFU of carboxyfluorescein succinimidyl ester (CFSE) surface-labeled *S*. *boulardii*, and sections of small intestine were harvested one hour later, embedded in optimal cutting temperature (OCT) compound, and cryosectioned as previously described [30]. Sections were stained with VECTASHIELD anti-fade mounting media with DAPI (4′,6-diamidino-2-phenylindole).

### ELISA

Assays for total antibody were performed by coating 96 well flat bottom MaxiSorp plates (Thermo Scientific) with unlabeled goat anti-mouse IgA and IgG (Southern Biotech) (S1 Table) diluted in carbonate/bicarbonate buffer overnight at 4°C. Alternatively, plates were coated with 10^7^ CFU heat-killed *S*. *boulardii* resuspended in carbonate/bicarbonate buffer (5.4 mM Na_2_CO_3_, 8.6 mM NaHCO_3_, pH 9.6) overnight at 4°C to detect antigen specific antibodies. Plates were blocked with TBST (150 mM NaCl, 15 mM Tris HCl, 4.6 mM Tris base, 0.5% Tween 20, pH 7.6) 5% nonfat dry milk for 2 hr at room temperature (RT) prior to incubation of serially diluted samples and standards overnight at 4°C. Goat anti-mouse IgA and IgG HRP-conjugated (Southern Biotech) antibodies were incubated for 1.5 hr at RT prior to addition of Super AquaBlue ELISA Substrate (eBiosciences) and reading at 405 nm. Anti-*S*. *cerevisiae* antibody (Abcam) and rabbit anti-goat IgG HRP-conjugated antibody (Southern Biotech) were used as positive controls for antigen specific assays. Purified mouse IgG (Invitrogen) and IgA (BD biosciences) antibodies were used as standards.

### Flow Cytometry

Spleens, MLNs, and PPs were washed with complete Iscoves’ Modified Dulbecco’s Medium (cIMDM) (Iscoves’ Modified Dulbecco’s Medium with 10% heat inactivated FCS, 100 U/mL penicillin, 100 μg/mL streptomycin, 2 mM L-glutamine, 50 μM 2-mercaptoethanol, and 1 mM sodium pyruvate, all Life Technologies except FCS from PAA laboratories) and homogenized using filtration over a 40 μm cell strainer. Samples used for analysis of CFSE-labeled yeast were resuspended in FACS buffer (1X PBS (Life Technologies), 5 mM EDTA, 2% FCS) and assayed without further staining. For experiments identifying germinal center B cells and plasma cells, homogenized cells were distributed at 10^6^ cells per well in a v bottom plate and blocked with anti-CD16/32 (BD biosciences). Cells were surface stained with antibody cocktails diluted in FACS buffer for 30 minutes on ice. Antibodies used include CD19 APC, Gl7 FITC, CD45R (B220) Pacific Blue, CD138 PE, all obtained from Biolegend. The Zombie NIR fixable live dead stain was also used as per manufacturer (Biolegend) protocols. Plasma cell populations were identified by Zombie^-^ CD138^+^CD45R^int^ expression; germinal center cells were identified by Zombie^-^CD19^+^CD95^+^GL7^+^ expression (S1 Fig) [31]. For detection of anti-*S*. *boulardii* antibody, diluted serum and fecal samples were incubated with 10^6^ whole *S*. *boulardii* for one hour at room temperature, followed by a 30 minute incubation with secondary goat anti-mouse IgA FITC (abcam) or donkey anti-mouse IgG PE (eBiosciences) and washes with FACS buffer. Stained cells were fixed with 2% paraformaldehyde and read on a BD LSR II flow cytometer. Analysis was conducted using FACS Diva and FlowJo software.

### ELISPOT

Millipore Multiscreen-HA 96-well plates (Millipore #MAHA N4510) were coated with anti-mouse IgG, IgA, IgM (Rockland) diluted to 5 μg/mL in PBS and incubated overnight at 4°C. Plates were then washed with PBST (1X PBS, 0.05% Tween 20) and PBS (1X, Life Technologies) (1x PBST, 3x PBS washes) and blocked by 2 hr incubation at 37°C with cIMDM. Media was then replaced with fresh cIMDM, and counted cells from spleens, MLN, and PP were added. Plates were incubated overnight at 37°C and, following washes (4x PBS, 4x PBST), biotin-conjugated anti-mouse IgG and IgA antibodies (Southern Biotech) were added at a concentration of 0.5 μg/mL diluted in PBST 1% FCS and incubated overnight at 4°C. Plates were washed (4x PBST) before incubation with a 1:1000 dilution of HRP avidin D (Vector Laboratories) in supplemented PBS (1X PBS, 0.05% Tween 20, 1% FCS) for 1–3 hr at room temperature. After washes (3x PBST, 3x PBS), AEC substrate (0.3mg 3-amino-9-ethylcarbazole in 0.1 M Na-Acetate buffer, pH 5, 0.3% hydrogen peroxide) was added and color reactions were allowed to proceed for 2–10 minutes before washing with distilled water. Plates were kept in the dark to dry until read and counted with the aid of a CTL ImmunoSpot 5.1.36 analyzer.

### RNA-sequencing

RNA extraction of MLNs from naïve and *S*. *boulardii*-treated C57BL/6J mice was performed using the Qiagen RNeasy mini kit with DNase treatment according to manufacturer’s protocols. Sample quality analyses, library preparation, and sequencing were performed by the Huntsman Cancer Institute High Throughput Genomics Core (University of Utah). RNA integrity was confirmed using an Agilent RNA ScreenTape assay, and only high quality RNA (RIN >8.0) was submitted for further processing. Library preparation with oligo dT selection was performed using the Illumina TruSeq Stranded mRNA Sample Preparation Kit. Sequencing libraries (25 pM) were chemically denatured and applied to an Illumina HiSeq v4 single read flow cell using an Illumina cBot. Single end sequencing of 50 bp reads was performed using an Illumina HiSeq 2000 according to standard protocols.

A mean of 41.1 million reads per sample were acquired, with very high quality as assessed by FastQC (Babraham Institute) (S2 Fig). Reads were mapped to the GRCm38 *Mus musculus* genome (accessed via *Ensembl* [23]) using TopHat2 [32]. HT-seq [33] count was used to assign aligned reads to genes from the *Ensembl* release 82 GRCm38 genome annotation. Differential expression analysis, MA plots, and clustering were performed with DESeq2 [34]. Genes with a p-value (adjusted for multiple corrections) of 0.05 or less were considered differentially expressed. Principal component analysis was performed with two components on the log-transformed expression of the 1,000 genes with highest variance among samples using the R package psych. RNA-seq reads have been deposited to the NCBI Sequence Read Archive (SRA) under accession number SRP067985.

## Results

### *S*. *boulardii* MYA-797 is genomically distinct from *S*. *cerevisiae*

**Fig 1:**
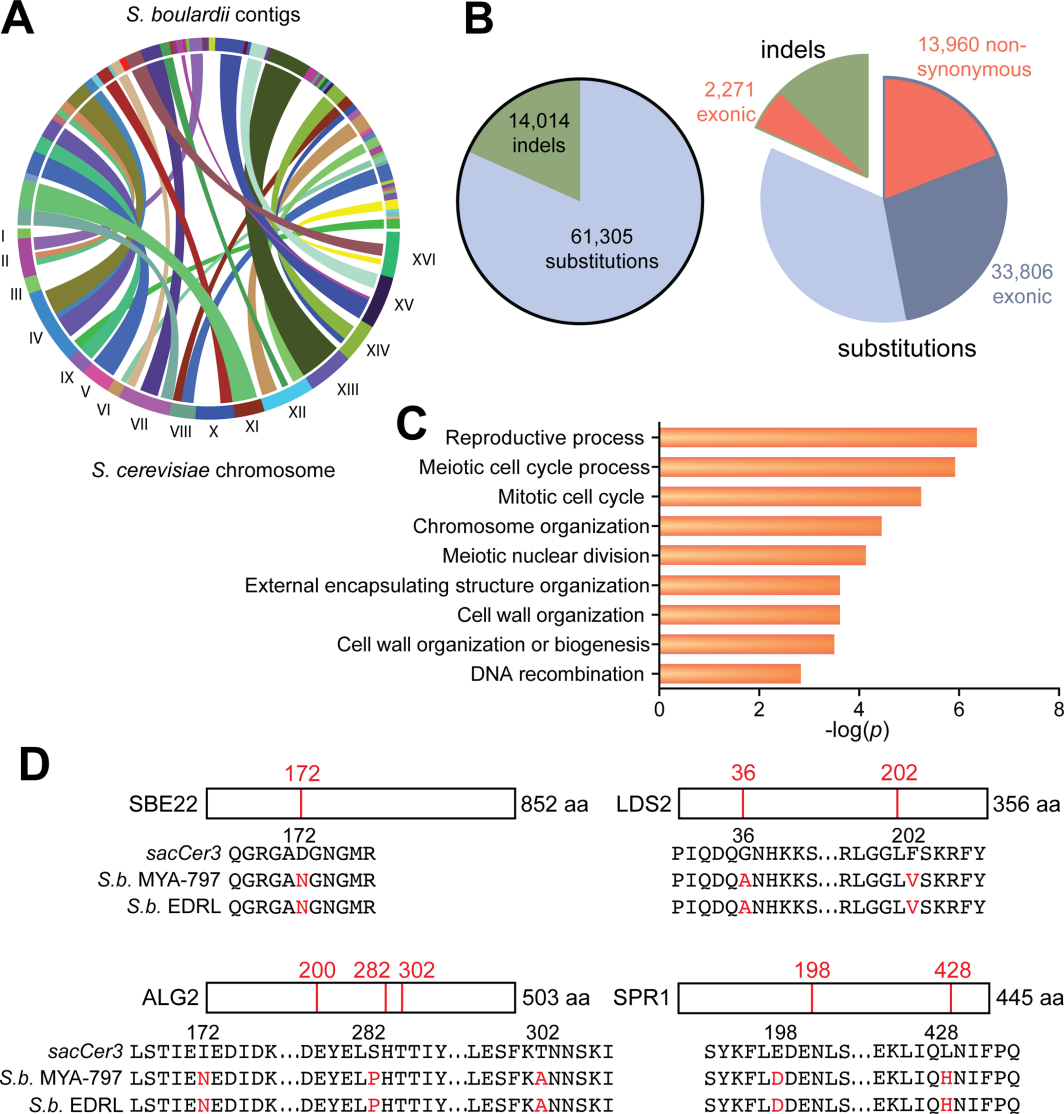
Sequencing of the *S*. *boulardii* genome reveals changes in genes involved in cell wall organization. (A) The *S*. *boulardii* ATCC MYA-797 genome was sequenced, yielding an 11.5 Mbp draft genome with 135 contigs of 1000 bp or more. Shown is a circle plot depicting synteny between the draft genome contigs and the *S*. *cerevisiae* sacCer3 reference genome. (B) Summary of sequence differences between the *S*. *boulardii* draft genome reported here and the *S*. *cerevisiae* reference genome. (C) Gene ontology analysis reveals that differences between *S*. *boulardii* and *S*. *cerevisiae* coding regions occur in genes critical for cell wall formation. Selected ontology terms and their Holm-Bonferroni *p*-values are shown. (D) Examples of the amino acid substitutions in the coding regions of *SBE22* [37], *ALG2* [38], *LDS2* [39], and *SPR1* [40], which all play important roles in cell wall formation.

*S*. *boulardii* has therapeutic traits that are distinct from many other *S*. *cerevisiae* strains [35]. Furthermore, experiments with probiotic bacteria demonstrate that effects of probiotics may differ depending on the strain and even isolate [36]. To explore genomic differences of the *S*. *boulardii* isolate here relative to *S*. *cerevisiae* and other known *S*. *boulardii* isolates [25], we performed genomic sequencing of *S*. *boulardii* ATCC MYA-797. Hiseq Illumina sequencing of *S*. *boulardii* genomic DNA provided a total of 105,329,454 paired end reads that were assembled using Velvet v1.2.10 into 424 total contigs, including 135 contigs of 1000 bp or more, to provide a draft genomic sequence of 11.5 Mbp with approximately 80x coverage. We identified numerous insertions/deletions (indels) and substitutions between *S*. *boulardii* ATCC MYA-797 contigs and the sacCer3 *S*. *cerevisiae* reference genome (Fig 1A and 1B). More than 16,000 of these changes are in exonic regions and encode amino acid substitutions. Gene ontology analysis of the genes with exonic indels and amino acid substitutions compared to *S*. *cerevisiae* revealed enrichment of numerous processes, including cell wall organization and assembly (Fig 1C, S2 Table). Alignment of sequences for genes important in cell wall formation, such as *SBE22* [37], *ALG2* [38], *LDS2* [39], and *SPR1* [40], of ATCC MYA-797 with both the sacCer3 *S*. *cerevisiae* reference genome and the previously published *S*. *boulardii* EDRL genome [25] reveals changes in coding regions leading to several amino acid substitutions shared by the two *S*. *boulardii* strains relative to *S*. *cerevisiae* (Fig 1D).

### The *S*. *boulardii* cell wall is thicker relative to *S*. *cerevisiae* strains and mediates stress resistance

**Fig 2:**
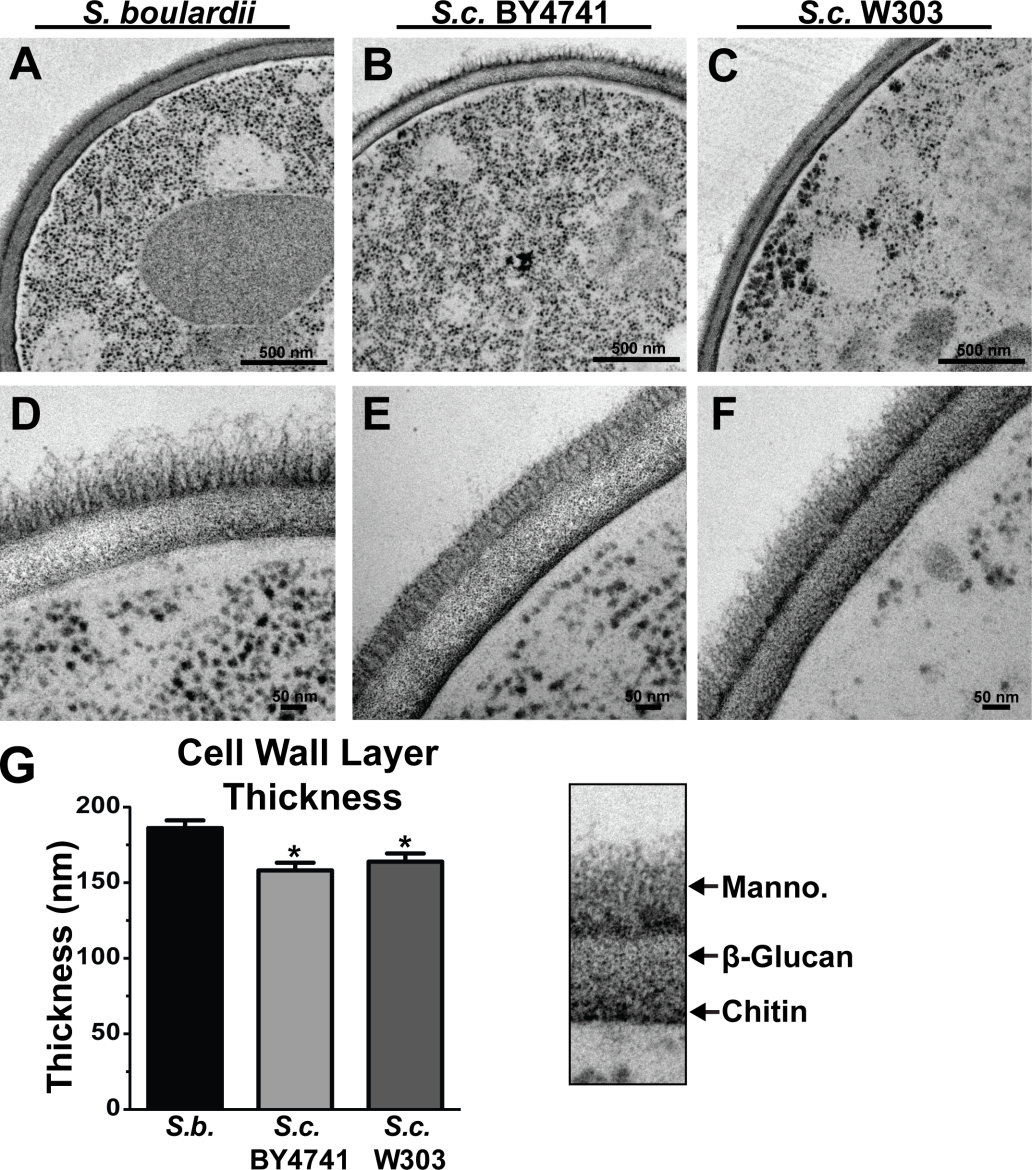
The cell wall of *S*. *boulardii* is thicker than in *S*. *cerevisiae* strains. *S*. *boulardii* (A, D) and *S*. *cerevisiae* BY4741 (B,E) and W303 (C, F) were cryopreserved and imaged via transmission electron microscopy. Scale bars denote 500 nm (A-C) and 50 nm (D-F). (G) Quantification of total cell wall thickness for each strain was calculated taking the average of 23 cells per strain. Error bars depict the standard error of the mean (SEM), * p <0.05 relative to *S*. *boulardii*, Kruskal-Wallis with Dunn’s multiple comparison test.

As sequencing of the *S*. *boulardii* genome revealed differences compared to *S*. *cerevisiae* in genes encoding proteins involved in cell wall formation, we compared the *S*. *boulardii* MYA-797 cell wall with two commonly used, well-characterized laboratory *S*. *cerevisiae* strains: BY4741 and W303. These strains were cryopreserved and imaged using transmission EM to visualize the cell wall (Fig 2). Images at low (Fig 2A–2C, scale bar 500 nm) and high (Fig 2D–2F, scale bar 50 nm) magnification reveal similar cell wall architecture among the studied strains. Although the major components of the yeast cell wall are integrated and not purely confined to specific lateral bands, regions of differing electron density identify cell wall layers composed primarily of these different components, namely a thin inner chitin layer, an internal β-glucan layer, and an outer mannoprotein layer [11]. Interestingly, the overall thickness of the *S*. *boulardii* cell wall is greater than for the two *S*. *cerevisiae* strains (Fig 2G), though this does not result from obvious increased thickness in any single cell wall layer relative to other strains. These differences in thickness and composition of the *S*. *boulardii* cell wall may account for some of the unique probiotic properties found for *S*. *boulardii* but not laboratory *S*. *cerevisiae* strains.

**Fig 3:**
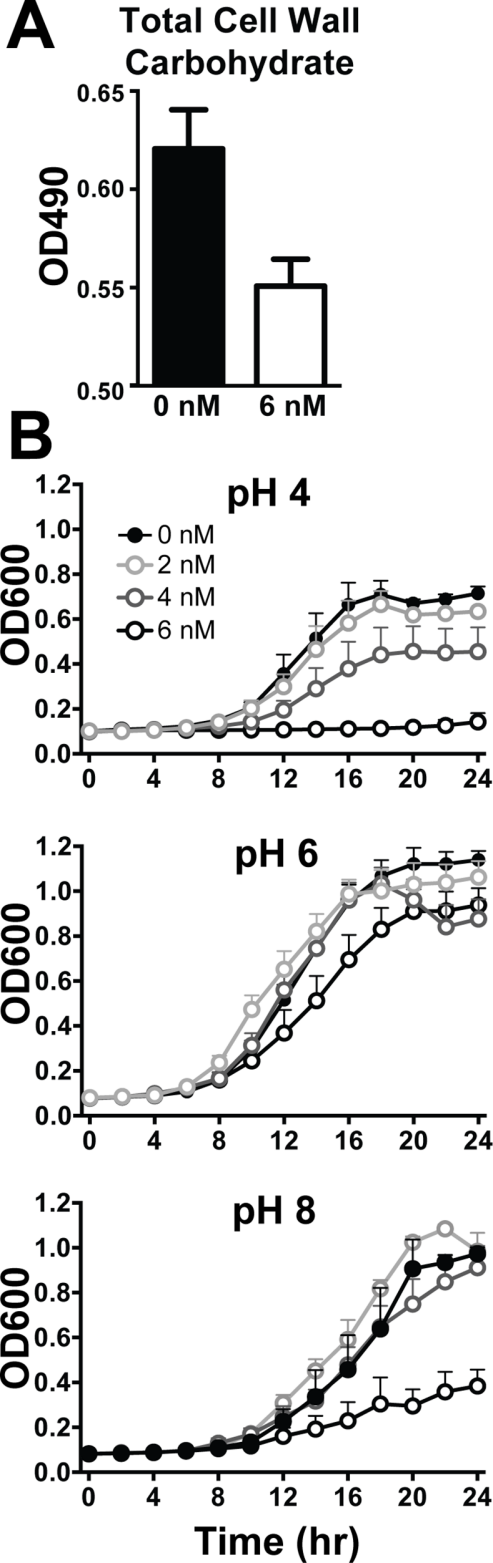
Caspofungin treatment alters *S*. *boulardii* cell wall structure and decreases resistance to pH. (A) To assess cell wall integrity,10^9^ yeast grown to saturation in either normal YPD media (0 nM) or media containing 6 nM caspofungin (6 nM) were tested using the phenol sulfuric acid assay, with higher OD_490_ readings indicating greater overall cell wall carbohydrate content. (B) Growth of *S*. *boulardii* in the presence of varying concentrations of caspofungin (0–6 nM) in normal media at pH 6 or media adjusted to pH 4 and pH 8 was measured by optical density (OD_600_) readings of cultures at the indicated times.

Previous studies have found that particular cell wall components, including β-glucans, increase resistance of probiotic bacteria to pH stresses and simulated gastrointestinal conditions [41]. To examine the role of the yeast cell wall in resistance to external stresses, *S*. *boulardii* was treated with caspofungin and exposed to media adjusted to pH levels that would be encountered in the digestive tract. Caspofungin is an echinocandin antifungal agent that inhibits yeast (1,3)-β-D-glucan synthase [42]. Use of the phenol sulfuric acid assay, a colorimetric assay to detect total monosaccharide content, showed that, as expected, treatment with caspofungin decreased *S*. *boulardii* total cell wall carbohydrate content (Fig 3A). Interestingly, although even the highest tested concentration of caspofungin only marginally decreased growth in normal media at pH 6, growth of caspofungin-treated *S*. *boulardii* was significantly impaired in the presence of media adjusted to pH 4 and pH 8 relative to untreated *S*. *boulardii* grown at the same pH (Fig 3B). This data shows that the integrity of the cell wall is important for the resistance of *S*. *boulardii* to fluctuations in pH that would be encountered as it passes through the gastrointestinal tract.

### Association and uptake of *S*. *boulardii* into small intestinal Peyer’s patches are low frequency events

**Fig 4:**
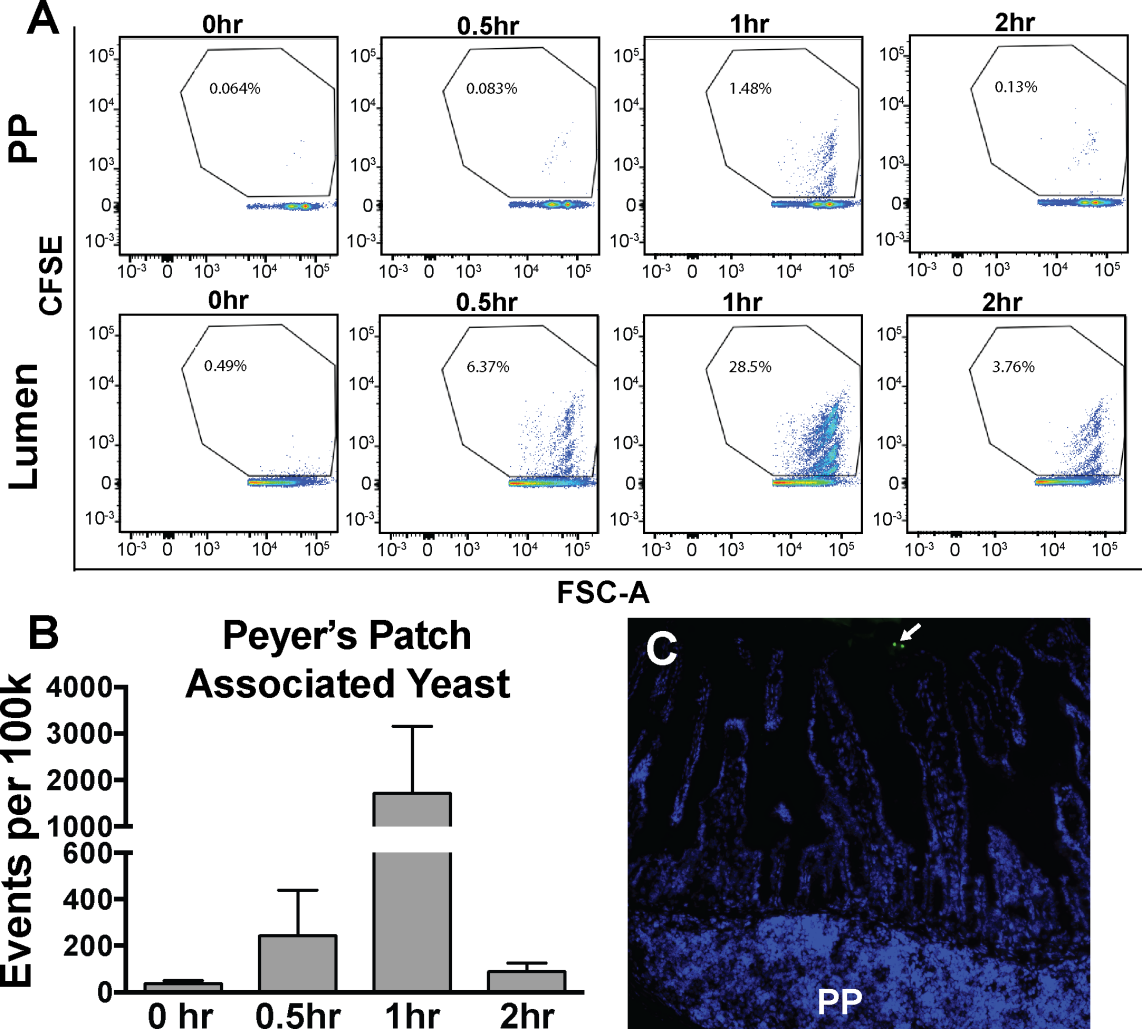
*In vivo* contact of *S*. *boulardii* with murine Peyer’s patches is limited. (A) C57BL/6 mice were gavaged with 10^8^ CFSE surface-labeled *S*. *boulardii*. Peyer’s patches (PP) and overlying luminal contents (Lumen) were collected 0, 0.5, 1, and 2 hours post gavage and analyzed by flow cytometry to detect CFSE^+^ events, as shown in representative flow plots. (B) Quantification of CFSE^+^ events per 100,000 cells in PP samples at each time point shows greatest association of yeast with these immune tissues at one hour post gavage (n = 4 PP per mouse in each of two independent experiments; error bars depict the standard error of the mean). (C) Immunohistochemistry showing CFSE-labeled *S*. *boulardii* (arrow) is largely excluded from contact with intestinal epithelial cells (DAPI) and a small intestinal Peyer’s patch (PP).

In order for any differences in cell wall composition to impact the ability of *S*. *boulardii* to deliver antigens and induce immune responses to therapeutics, the yeast must be able to contact immune cells in the intestine. To determine the degree to which *S*. *boulardii* is able to contact and adhere to antigen sampling sites, PPs from C57BL/6 mice were harvested at multiple time points after gavage with single doses of carboxyfluorescein succinimidyl ester- (CFSE) labeled *S*. *boulardii*. As shown in representative flow cytometry plots (Fig 4A), labeled yeast were clearly detected in PP samples collected one hour after gavage. Samples of luminal contents overlying collected PPs were also assayed as positive controls and demonstrate presence of yeast in the intestinal lumen at each time point after gavage, peaking at 1 hr. Quantification (Fig 4B) of CFSE+ events in PP samples at each time point shows the greatest degree of association at 1 hr.

While the presence of CFSE positive events in these samples suggests association of yeast with PPs, these numbers are low relative to the initial inoculum and do not demonstrate uptake of yeast into the PPs themselves. Histological evaluation of intestinal sections from mice gavaged with CFSE-labeled *S*. *boulardii* further demonstrate the low frequency of intact yeast near the epithelium (Fig 4C). Indeed, no yeast were detected immediately adjacent to or within PPs using this approach.

### *S*. *boulardii* induces marginal increases in total, but not antigen specific, antibody levels

The large quantities of IgA secreted into the intestinal lumen form a critical component of intestinal homeostasis and defense against invading pathogens [43]. Indeed, pathogen-specific antibody titers are the gold standard in measuring responses to vaccine, and IgA antibody titers are often used as an indicator of protection against mucosal diseases. A reported feature of the mucosal immune response to *S*. *boulardii* is increased total secretory IgA (sIgA) levels [20–22], although these studies were conducted in gnotobiotic and weanling rodent models which are known to have differences in B cell responses and antibody levels relative to specific-pathogen-free (SPF) mice [44]. To determine the nature of *S*. *boulardii*-induced antibody production in adult SPF mice and to determine if prolonged exposure to *S*. *boulardii* further increases antibody levels over time, C57BL/6 mice were orally gavaged with 10^8^ CFU *S*. *boulardii* or control vehicle daily for 7, 14, or 28 days, and sera and fecal samples were collected to determine antibody levels.

**Fig 5:**
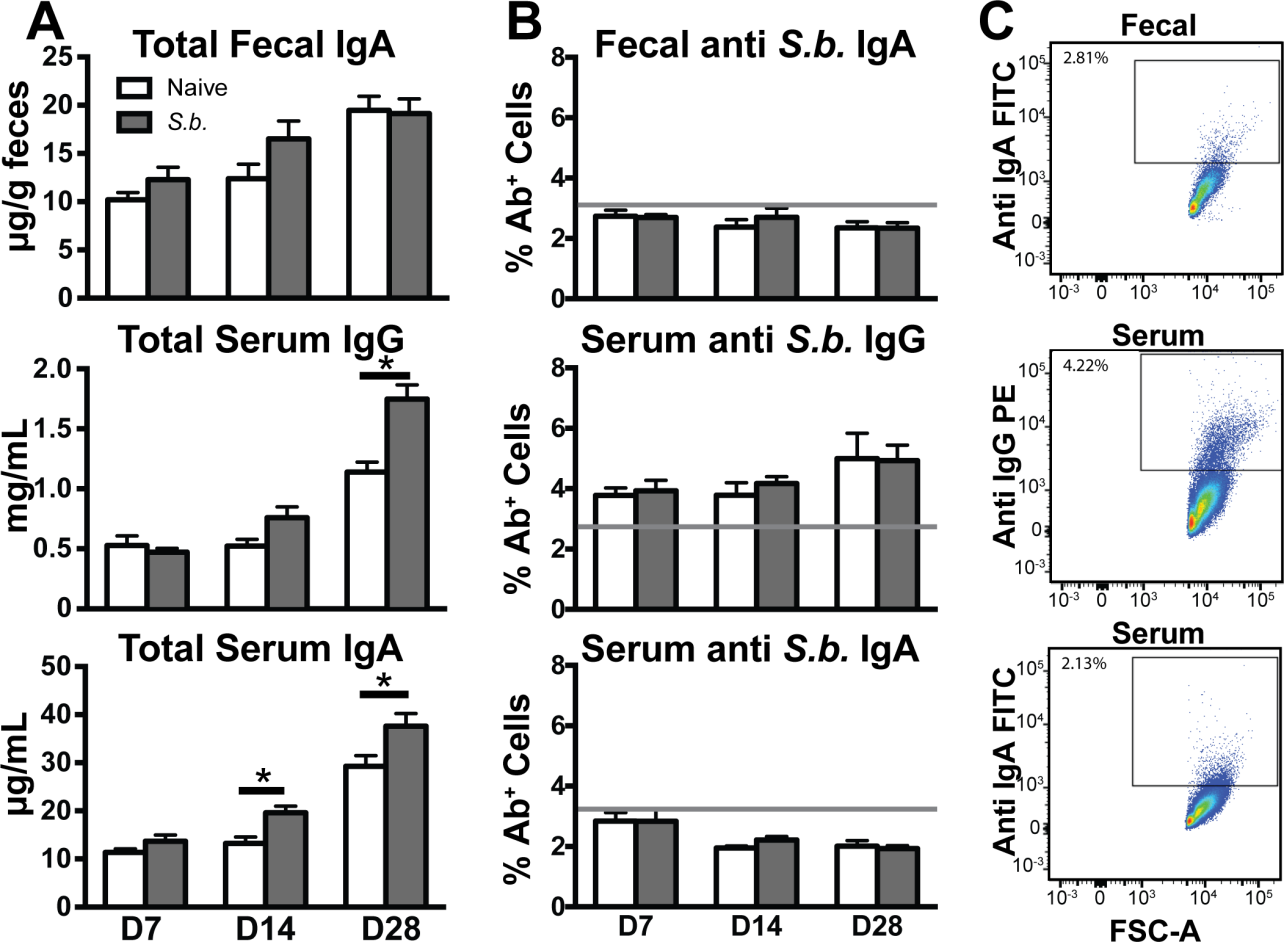
*S*. *boulardii* induces increased total but not antigen specific fecal and serum antibody levels. (A) Total fecal IgA, serum IgG, and serum IgA levels were determined by ELISA using samples from mice gavaged with vehicle (white bars) or 10^8^ CFU *S*. *boulardii* (gray bars) daily for 7, 14, or 28 days (n = 5 mice per group in each of two independent experiments per time point, with error bars showing the standard error of the mean (SEM), *p<0.05, ordinary two-way ANOVA, Sidak multiple comparison test). (B) Percentage of *S*. *boulardii* cells positive for IgA or IgG after incubation with serum or fecal samples collected from naïve mice (white bars) or mice gavaged daily with *S*. *boulardii* (gray bars) for 7, 14, or 28 days, with gray lines showing the average percentage of stained cells in control samples incubated with secondary antibody only (n = 5 mice per group in each of two independent experiments. Error bars depict SEM.) (C) Representative flow plots depicting the percent of total *S*. *boulardii* cells positive for IgA or IgG after incubation with either serum or fecal supernatant.

As expected, total fecal IgA levels as determined by ELISA increase in *S*. *boulardii-*treated mice relative to naïve mice until day 28, although this did not reach statistical significance (Fig 5A). *S*. *boulardii*-treated mice also showed increases in total serum IgG and IgA, suggesting that oral gavage with *S*. *boulardii* induces some degree of systemic immune effects, although limited. *Ex vivo* incubation of *S*. *boulardii* with serum and fecal supernatant collected from naïve and *S*. *boulardii-*gavaged mice furthermore enabled detection of *S*. *boulardii*-reactive antibody by flow cytometry (Fig 5B and 5C). The percent of *S*. *boulardii* opsonized with antibody did not increase when cells were incubated with samples from *S*. *boulardii*-gavaged mice relative to samples from control mice, indicating no increase in anti-*S*. *boulardii* antibody levels in treated mice. Analysis by ELISA also demonstrated no detectable levels of *S*. *boulardii*-reactive antibody in either group (S3 Fig). These findings indicate that although *S*. *boulardii* induces both a local and systemic increase in total antibody, this response requires numerous doses to reach significance and does not induce antibodies reactive against yeast antigens themselves.

### *S*. *boulardii* induces limited changes in numbers of germinal center B cells and plasma cells

**Fig 6:**
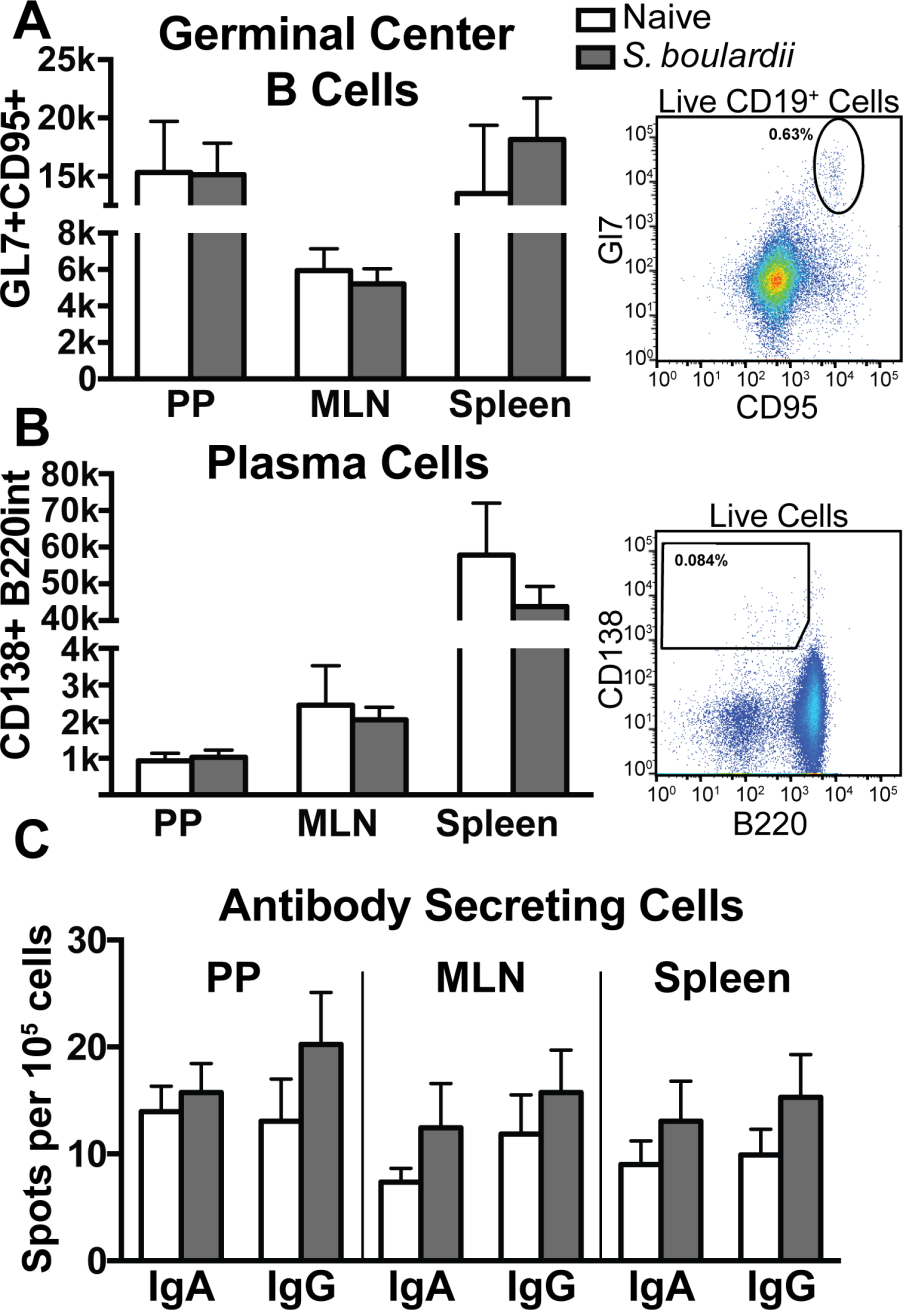
*S*. *boulardii-*gavaged mice show only marginal differences in B lineage cell populations. Peyer’s patches (PP), mesenteric lymph nodes (MLNs), and spleens were collected from mice gavaged daily with vehicle (white bars) or 10^8^ CFU *S*. *boulardii* (gray bars) for 28 days and analyzed by flow cytometry and ELISPOT to analyze *S*. *boulardii*-induced changes in B cell lineage cells. Representative flow plots and quantification of total numbers of germinal center B cells (CD19^+^GL7^+^CD95^+^) (A) and plasma cells (CD138^+^B220^int^) (B) show no statistically significant differences in these populations (n = 5 mice per group in each of two independent experiments). (C) Spots per 5x10^5^ cells indicate the number of IgA or IgG secreting cells in each tissue, as determined by ELISPOT. Error bars depict SEM.

To further investigate the nature of B cell responses to *S*. *boulardii*, mice were gavaged with daily doses of vehicle or 10^8^ CFU *S*. *boulardii* for 28 days. Peyer’s patches (PP), mesenteric lymph nodes (MLN) and spleens were assayed for numbers of germinal center B cells (C19^+^Gl7^+^CD95^+^) (Fig 6A) and plasma cells (CD138^+^B22^int^) (Fig 6B) by flow cytometry (gating strategy shown in S1 Fig). Quantification indicated no significant differences in the number of germinal center B cells or plasma cell in the PPs, MLNs, or spleens of *S*. *boulardii*-treated versus naïve mice. Total cell numbers of each tissue were not significantly different between groups, and cell percentages reflected similar patterns seen by cell number (S4 Fig). Thus there are only minimal differences induced in B cell populations by *S*. *boulardii* in the healthy immune system.

### *S*. *boulardii* induces trends toward increased numbers of antibody secreting cells

To further analyze antibody responses to *S*. *boulardii*, ELISPOT analysis of PPs, MLNs, and spleens harvested from mice after 28 days of gavage was used to enumerate antibody secreting cells in these tissues. Although IgA and IgG secreting cells showed consistent trends toward increased numbers in *S*. *boulardii-*treated mice, none of these differences reached statistical significance (Fig 6C).

### *S*. *boulardii* induces minimal changes in MLN gene expression

To quantify gene expression changes in other immune pathways induced by *S*. *boulardii* treatment, we isolated RNA from whole MLNs of mice gavaged daily with 10^8^ CFU of *S*. *boulardii* or PBS. RNA from two mice in each group was sequenced, for a total of 164 million reads. Reads were aligned to the GRCm38 mouse genome and gene expression changes were calculated using DESeq2 (http://www.genomebiology.com/content/15/12/550). 19,601 total genes were identified with 5 or more normalized counts.

**Fig 7:**
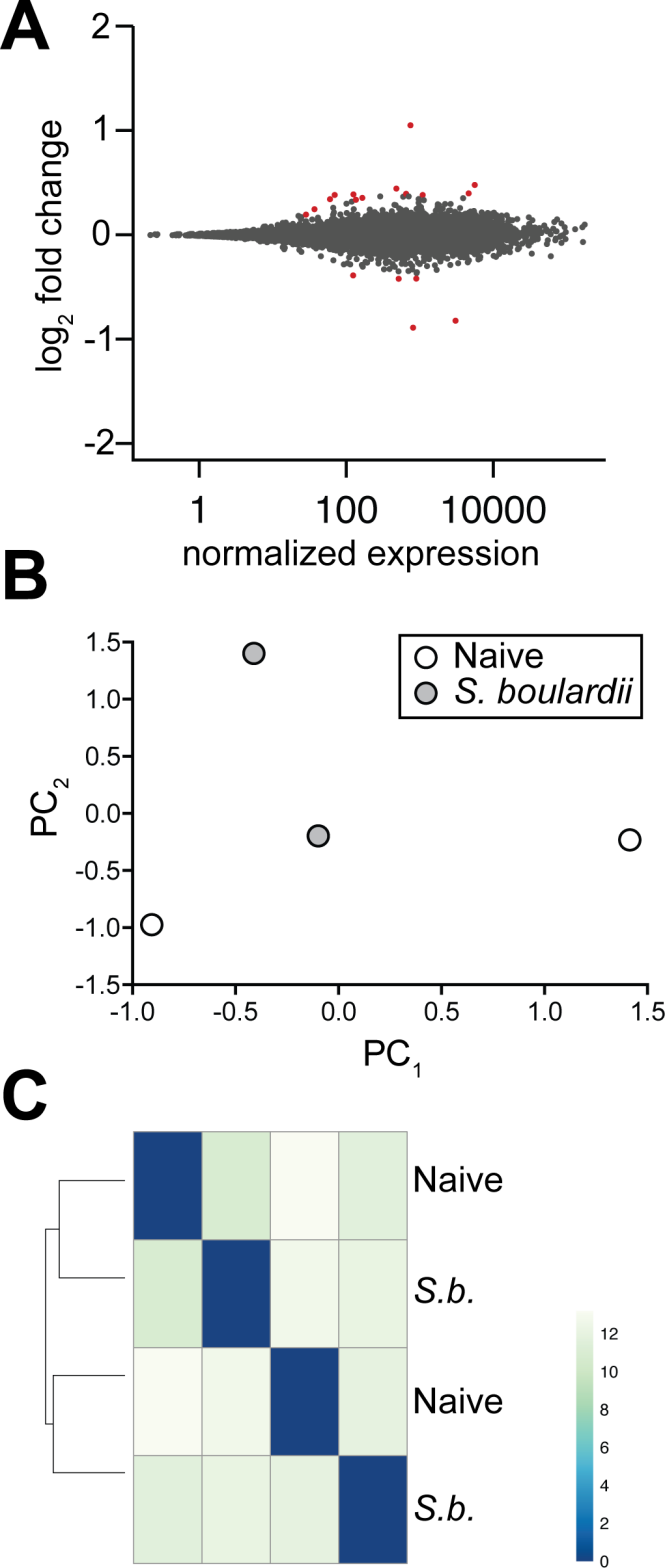
RNA-sequencing of MLNs reveals few differences in gene expression between *S*. *boulardii*-treated and naïve mice. (A) MA plot depicting log scale average gene expression versus fold change of gene expression in *S*. *boulardii*-treated versus naïve mice on the x and y axes, respectively. Differentially expressed genes are shown in red. (B) Principal component analysis and (C) sample-to-sample distance clustering of naïve and *S*. *boulardii*-treated samples demonstrate no large effect of *S*. *boulardii* on gene expression in mouse MLNs.

Overall, expression of very few genes changed significantly with *S*. *boulardii* treatment versus vehicle (Fig 7A, S3 Table); only fourteen genes were identified as differentially expressed (p-value adjusted for multiple comparisons < 0.05) between the two groups. Both principal component analysis (Fig 7B) and clustering analysis (Fig 7C) demonstrate that gene expression differences detected are driven by differences between individual mice rather than any differences induced by *S*. *boulardii* treatment.

## Discussion and Conclusions

Probiotic organisms provide clinical benefits in the context of numerous infectious and inflammatory gastrointestinal disorders. However, current prophylactic use of these probiotics [45] as well as their potential application for delivering therapeutics to the gastrointestinal tract [1,2,46] necessitates an understanding of how these organisms interact with the uninflamed, healthy mucosa. Probiotic organisms may exert beneficial effects through modulation of the immune system, yet the immunogenicity of probiotics within the healthy intestine is not well described. In this study, we have assessed intrinsic properties and immunomodulatory interactions of the probiotic yeast *S*. *boulardii* in the healthy intestine.

Previous studies have reported that *S*. *boulardii* administered to mice has immunomodulatory effects such as induction of antibodies [20–22]. In our hands, *S*. *boulardii* does not invoke a significant immune response in the context of the healthy gastrointestinal tract (Figs 5 and 6). However, we have identified a number of genomic and phenotypic differences between *S*. *boulardii* and non-probiotic *S*. *cerevisiae* that may explain some of the probiotic effects of *S*. *boulardii* in disease states. For example, cell wall thickness of *S*. *boulardii* was noticeably greater compared to *S*. *cerevisiae* (Fig 2), and this is consistent with genomic differences in genes encoding proteins involved in cell wall function (Fig 1).

Although no *S*. *boulardii-*reactive antibody was detectable even after multiple doses of *S*. *boulardii*, we did observe statistically significant, albeit marginal, increases in serum IgA and IgG (Fig 5 and S3 Fig). These data are consistent with previous studies showing no effect on the number of antibody-producing B cells in human peripheral blood mononuclear cells (PBMCs) upon consumption of *S*. *boulardii* (31 and 32). Our studies in mice enabled a more detailed, *in vivo* analysis of localized immune responses in the gastrointestinal tract. However, we did not detect any significant changes in antibody levels or B cell populations in the mucosa (Figs 5 and 6). An extended analysis to evaluate potential effects of *S*. *boulardii* on other immune cell pathways by performing RNA-seq of the mesenteric lymph nodes further confirmed the minimal impact of *S*. *boulardii* on the local mucosal immune response (Fig 7). The minimal number of gene expression changes detected in this analysis suggest that administration of *S*. *boulardii* in the context of the healthy mucosa does not induce an inflammatory immune response and has little effect in potentiating B and T cell responses.

Results of the present study are consistent with a recent report that the probiotic bacterium *Lactobacillus rhamnosus* GG influences the T helper cell balance in Crohn’s disease patients but not in healthy control patients [4]. These findings led to the suggestion that effects of probiotics are limited in the healthy intestine, possibly due in part to restricted contact between probiotics and immune cells. However, in the inflamed intestine, disruption of barrier integrity, microbiota changes, and recruitment of inflammatory cells likely all increase the frequency of encounters between probiotics and immune cells. Our data strongly suggest that in the healthy adult mouse intestine the majority of *S*. *boulardii* do not contact the gastrointestinal epithelium, including the Peyer’s patches of the small intestine (Fig 4). If uptake of whole intact yeast is indeed a low frequency event, use of *S*. *boulardii* as a vaccine delivery vector may require optimization to increase efficiency of antigen delivery to immune cells underlying the epithelium.

Several approaches may permit increased contact of *S*. *boulardii* with the epithelium and improve therapeutic delivery. Studies examining use of probiotic bacteria as delivery vehicles have proposed heterologous expression of M cell ligands to increase contact and uptake of probiotics by these antigen sampling cells on Peyer’s patches [48,49]. It is known that particle uptake by M cells is size-dependent [50], and in the case of *S*. *boulardii* (3–10 μm average diameter) uptake of whole yeast may be restricted. Inducing vaccine antigen secretion such as through use of the *S*. *cerevisiae* alpha mating factor prepro leader sequence may thus be needed to enable uptake by antigen sampling cells [46]. This approach in combination with expression under the control of promoters that are activated in response to the alkaline or low oxygen conditions of the small intestine [51], may prove beneficial for future applications using *S*. *boulardii* to deliver heterologous protein to the intestine. These features have already been explored for a strain of probiotic bacteria [52], and the genomic sequences of multiple *S*. *boulardii* isolates now available will facilitate identification and cloning of promoters for this probiotic yeast.

The limited immunomodulatory effects of *S*. *boulardii* in the healthy intestine have several implications for its use as a vaccine delivery vector as well as a prophylactic agent. Many studies have identified prophylactic and therapeutic efficacy of *S*. *boulardii* in rodents and humans. For example, *S*. *boulardii* treatment has been shown to decrease intestinal histological damage and death upon *C*. *difficile* challenge in mice [53], reduce bacterial translocation in mice challenged with *Salmonella enterica* serovar Typhimurium [54], and reduce the risk of antibiotic-associated diarrhea in humans [55]. Prophylactic efficacy may be due to local effects of *S*. *boulardii* within the intestine such as by contributing to maintenance of a normal microbiota [56] or affecting epithelial integrity, such as through trophic effects as have been previously described [47,57]. These beneficial attributes may help buffer the intestine against pathogenic or inflammatory challenge; however, results of the present study suggest that protective effects of *S*. *boulardii* in the healthy intestine are not immune-mediated. As a vaccine delivery vector, the limited immunomodulation of *S*. *boulardii* in the healthy intestine may allow for applications in a wide range of diseases. Such a vector could potentially be used to deliver vaccine antigen without inducing damaging inflammatory responses that would lead to rapid yeast clearance and prevent adequate therapeutic delivery. Furthermore, the lack of T helper cell polarization in response to *S*. *boulardii* could allow for use of specific adjuvants in combination with *S*. *boulardii* to arm particular immune effector mechanisms in a manner tailored to the pathogen of interest.

In summary, we present an investigation into both intrinsic and immunological properties of the probiotic yeast *S*. *boulardii* in the healthy intestine. We show that delivery of *S*. *boulardii* to mice is relatively benign with respect to the induction of a mucosal immune response, suggesting that *S*. *boulardii* may exert reported beneficial effects in the healthy gastrointestinal tract by mechanisms that are not based on immune system modulation. These findings inform future work using *S*. *boulardii* in the healthy intestine and provide rationale for better optimization and testing of *S*. *boulardii* as a vaccine delivery vector.

## Supporting Information

S1 Fig Gating strategy for B cell flow cytometry panel. Cell were gated first on lymphocytes, then single cells by FSC-H FSC-W and SSC-H SSC-W gates, and then Zombie NIR negative populations to determine live cells from which plasma cells were then gated. To determine germinal center B cells, live cells were further gated to identify the CD19^+^ population.
(TIF)

S2 Fig RNA-seq read quality and mapping statistics. (A) Per-sequence quality score (as determined by FastQC) of the four sequenced samples. (B) Bowtie2 alignment summary for each sample.
(TIF)

S3 Fig Anti-*S*. *boulardii* antibody levels as determined by ELISA are below detectable limits. Plates coated with heat-killed *S*. *boulardii* were used in ELISA to determine *S*. *boulardii* specific antibody levels in the feces (A) and serum (B,C) of naïve (white bars) and *S*. *boulardii*-treated (gray bars) mice. No antigen specific antibody levels were above detectable limits at days 7, 14, or 28. Limit of detection (solid line) was determined using a control anti-*Saccharomyces cerevisiae* antibody, and background level (dashed line) was determined using the OD_405_ readings of blank wells.
(TIF)

S4 Fig Cell numbers and percentages of plasma cells and germinal center B cells are not significantly different. (A) Numbers of total live cells as determined by hemocytometer counts with trypan blue staining show no difference in the size of PPs, MLNs, or spleens of *S*. *boulardii*-treated (gray bars) and naïve (white bars) mice. Percentages of germinal center B cells (B) and plasma cells (C) in each tissue as determined by flow cytometry are also not statistically different.
(TIF)

S1 Table Reagents and Antibodies. (DOCX)

S2 Table Gene Ontology Terms. Processes enriched among *S*. *boulardii* ATCC MYA-797 genes with exonic indels and amino acid substitutions compared to *S*. *cerevisiae* sacCer3, as determined by gene ontology analysis using the *Saccharomyces* genome database (http://www.yeastgenome.org/) [26].
(XLSX)

S3 Table DESeq2 Output. (XLSX)
