# Supplementary figures and images for: Characterization of the Probiotic Yeast Saccharomyces boulardii in the Healthy Mucosal Immune System
Source: PLoS One. 2016 Apr 11;11(4):e0153351. doi: 10.1371/journal.pone.0153351 (PMC4827847; doi:10.1371/journal.pone.0153351)

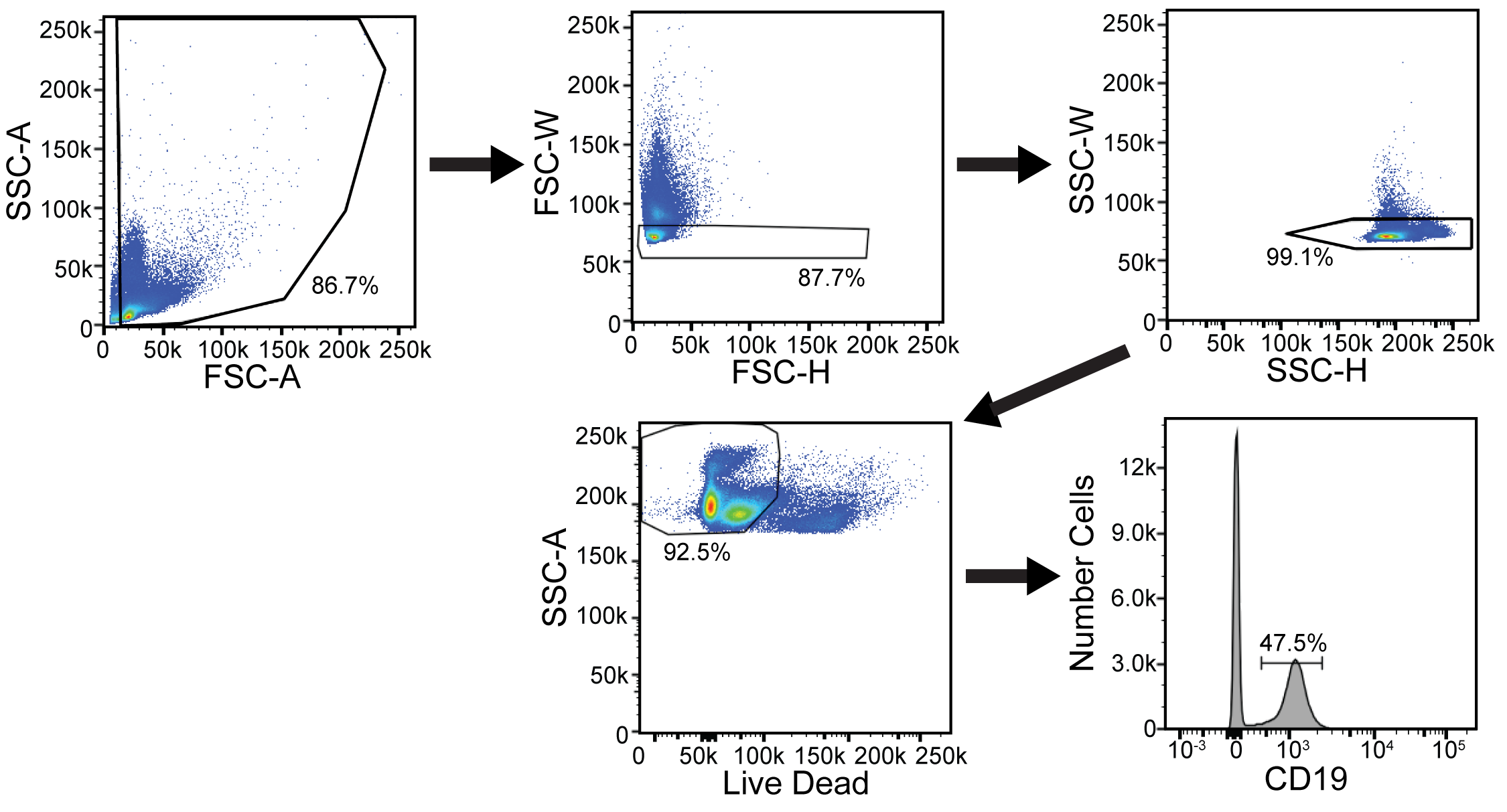

Supplement: S1 Fig — Cell were gated first on lymphocytes, then single cells by FSC-H FSC-W and SSC-H SSC-W gates, and then Zombie NIR negative populations to determine live cells from which plasma cells were then gated. To determine germinal center B cells, live cells were further gated to identify the CD19+ population. (TIF) [file pone.0153351.s001.tif]

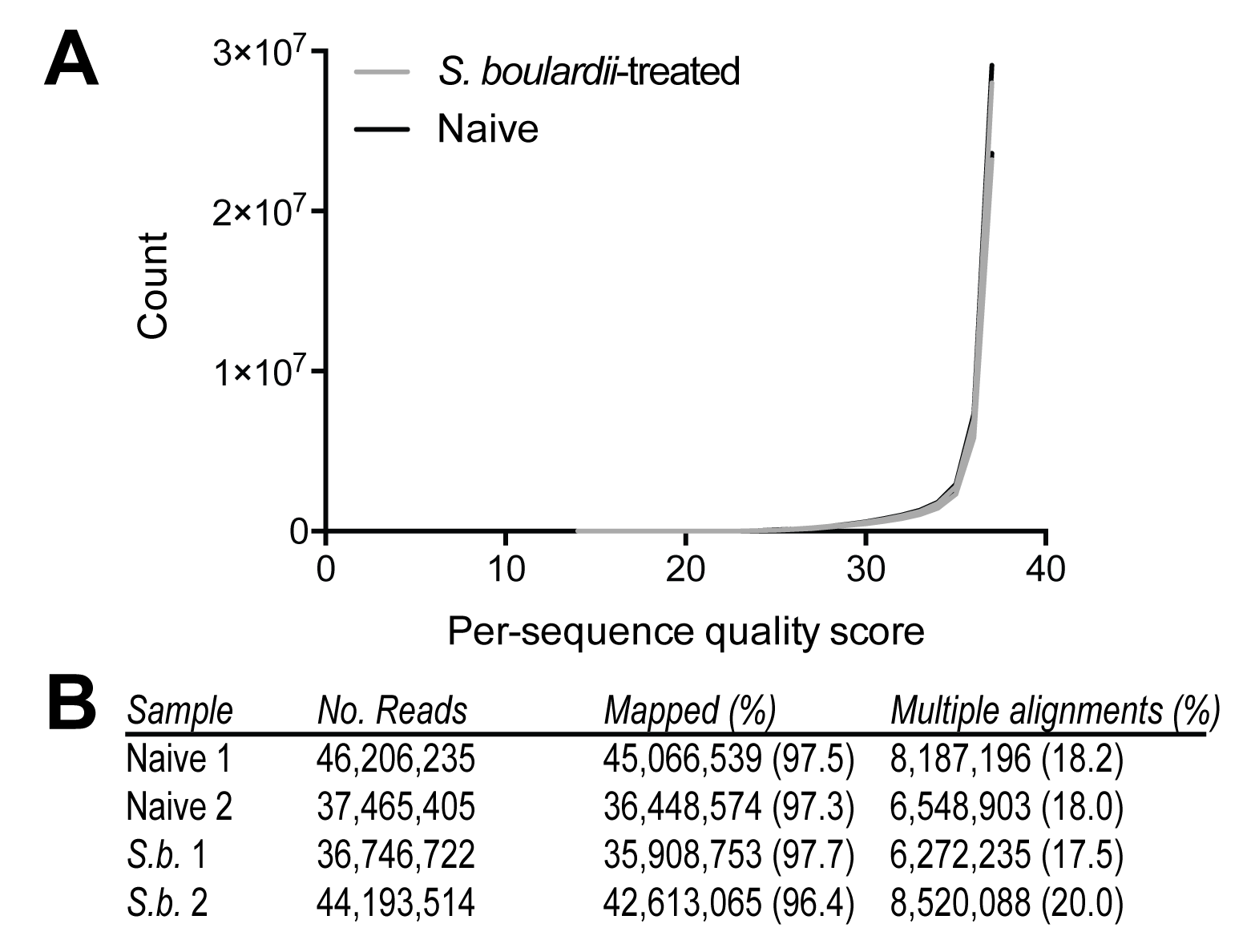

Supplement: S2 Fig — (A) Per-sequence quality score (as determined by FastQC) of the four sequenced samples. (B) Bowtie2 alignment summary for each sample. (TIF) [file pone.0153351.s002.tif]

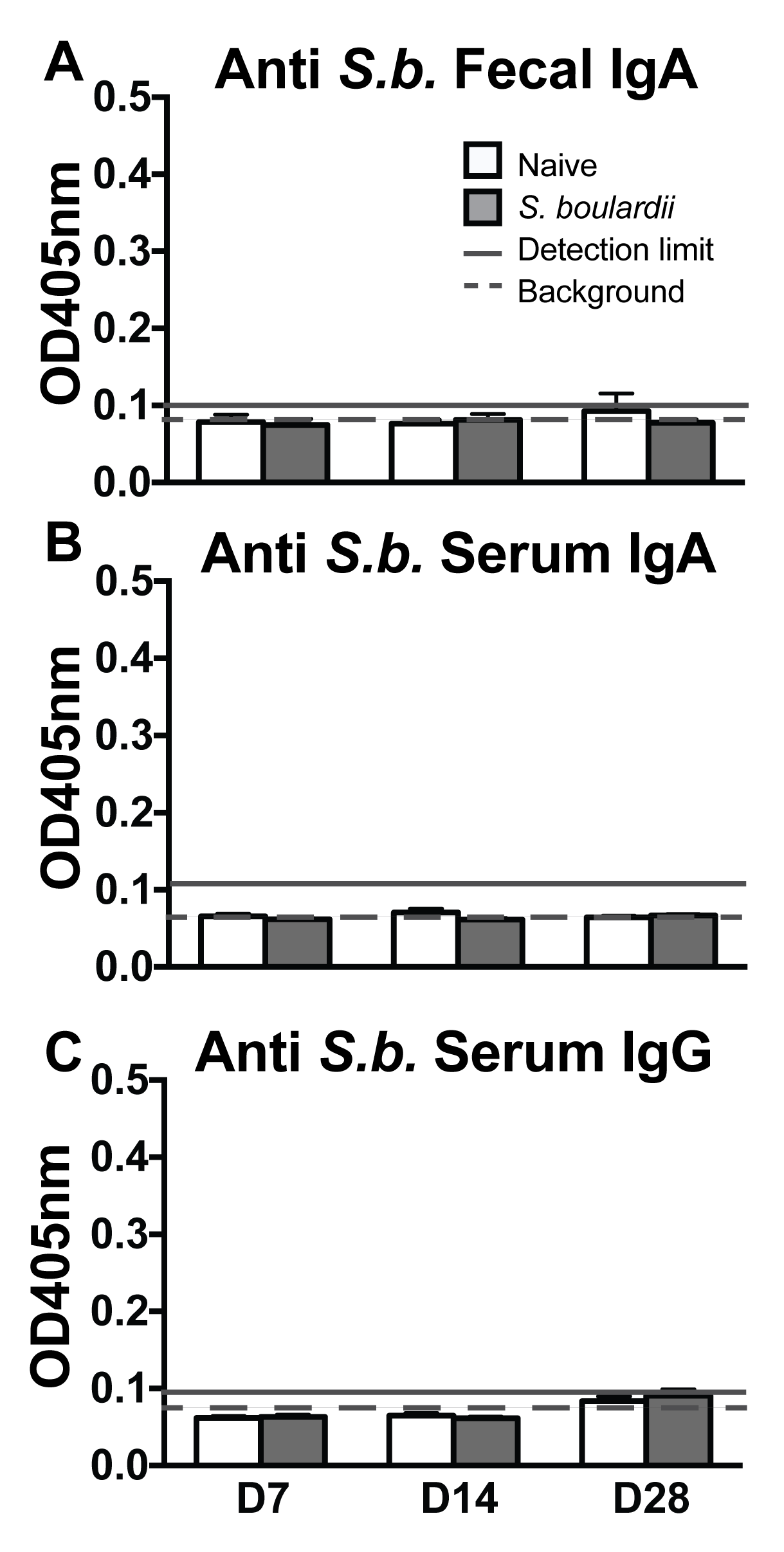

Supplement: S3 Fig — Plates coated with heat-killed S. boulardii were used in ELISA to determine S. boulardii specific antibody levels in the feces (A) and serum (B,C) of naïve (white bars) and S. boulardii-treated (gray bars) mice. No antigen specific antibody levels were above detectable limits at days 7, 14, or 28. Limit of detection (solid line) was determined using a control anti-Saccharomyces cerevisiae antibody, and background level (dashed line) was determined using the OD405 readings of blank wells. (TIF) [file pone.0153351.s003.tif]

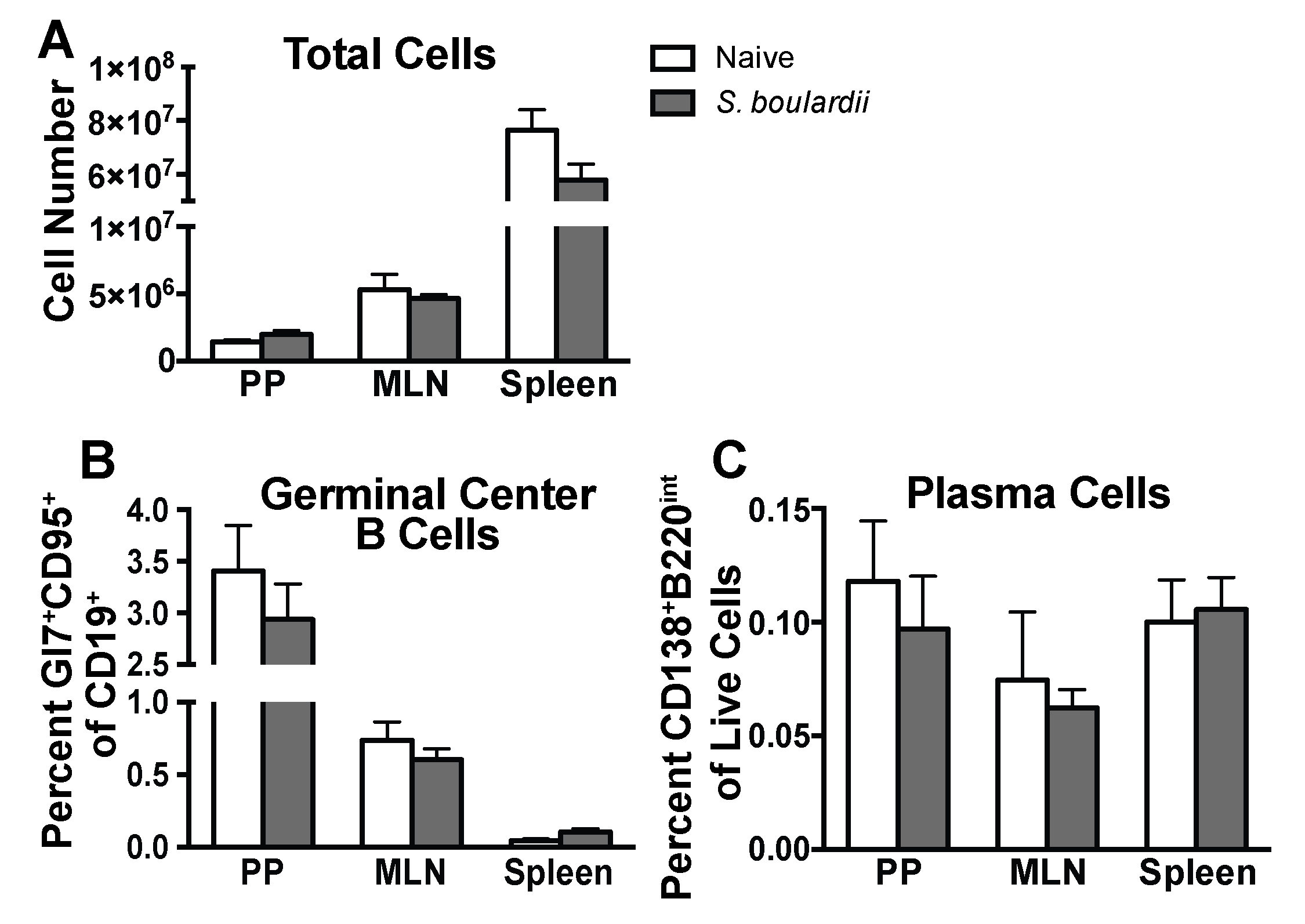

Supplement: S4 Fig — (A) Numbers of total live cells as determined by hemocytometer counts with trypan blue staining show no difference in the size of PPs, MLNs, or spleens of S. boulardii-treated (gray bars) and naïve (white bars) mice. Percentages of germinal center B cells (B) and plasma cells (C) in each tissue as determined by flow cytometry are also not statistically different. (TIF) [file pone.0153351.s004.tif]
