# Supplementary material for: Characterization of the Probiotic Yeast Saccharomyces boulardii in the Healthy Mucosal Immune System
Source: PLoS One. 2016 Apr 11;11(4):e0153351. doi: 10.1371/journal.pone.0153351 (PMC4827847; doi:10.1371/journal.pone.0153351)
Supplement: S1 Table — (DOCX) [file pone.0153351.s005.docx]

| **Reagent** | **Company** | **Catalog Number** |
| --- | --- | --- |
| PBS | Life Technologies | 10010-023 |
| P8340 protease inhibitor | Sigma | P8340 |
| Optimal cutting temperature (OCT) compound | Fisher Scientific | 4585 |
| Super AquaBlue ELISA Substrate | eBiosciences | 00-4203-58 |
| Iscoves’ Modified Dulbecco’s Medium | Life Technologies | 31980-030 |
| Zombie NIR fixable live dead stain | Biolegend | 423106 |
| HRP avidin D | Vector Laboratories | A-2004 |
| RNeasy mini kit | Qiagen | 74104 |
|  | | |
| **Antibody** | **Company** | **Catalog Number** |
| Unlabeled goat anti-mouse IgA | Southern Biotech | 1040-01 |
| Unlabeled goat anti-mouse IgG | Southern Biotech | 1030-01 |
| HRP-conjugated goat anti mouse IgA | Southern Biotech | 1040-05 |
| HRP-conjugated goat anti mouse IgG | Southern Biotech | 1030-05 |
| Anti-*S. cerevisiae* | Abcam | ab25813 |
| HRP-conjugated rabbit anti-goat IgG | Southern Biotech | 6160-05 |
| Purified mouse IgG | Invitrogen | 02-6502 |
| Purified mouse IgA | BD Biosciences | 553476 |
| anti-CD16/32 | BD Biosciences | 553142 |
| CD19 APC | Biolegend | 115512 |
| Gl7 FITC | Biolegend | 144604 |
| CD45R Pacific Blue | Biolegend | 103227 |
| CD138 PE | Biolegend | 142503 |
| Goat anti mouse IgA FITC | Abcam | ab97234 |
| Donkey anti mouse IgG PE | eBiosciences | 12-4012-82 |
| Anti mouse IgG, IgA, IgM | Rockland | 610-101-130 |
| Biotin-conjugated anti mouse IgG | Southern Biotech | 1030-08 |
| Biotin-conjugated anti mouse IgA | Southern Biotech | 1040-08 |
